# Supplementary material for: “I Genuinely Believe This Is the Most Stigmatised Group within the Social Care Sector”—Health and Social Care Professionals’ Experiences of Working with People with Alcohol-Related Brain Damage: A Qualitative Interview Study
Source: Int J Environ Res Public Health. 2023 Dec 20;21(1):10. doi: 10.3390/ijerph21010010 (PMC10815022; doi:10.3390/ijerph21010010)
Supplement: Supplementary file 1 [file ijerph-21-00010-s001.zip › ijerph-2730139-supplementary.pdf]

Supplementary Materials (Text S1)

Topic Guide

Introductory Questions

- Could you explain what this institution does?
- Could you explain what your role entails?

Topics

- What can you tell me about Alcohol Related Brain Damage?
    - Diagnosis?
    - Symptoms?
- Do you encounter people with this diagnosis?
    - What is your experience with this diagnosis?
    - Have you made an ARBD diagnosis?
    - If you have made such a diagnosis how often was this?
- Can you tell me more about the care and treatment a person with this diagnosis?
    - Do you provide this care/treatment?
    - Where can you refer this person to?
    - Do you know any other organisations that work with this diagnosis?
- Do you enquire about a patient's alcohol use?
    - Why/why not?
    - What is done with this information?
- Sum up what has been discussed.
    - Is there anything important to you we haven't mentioned?
    - If you want to follow up on any issues you have talked about, I would like to refer you to the debrief-sheet.
